# Two new species of *Lobrathium* Mulsant & Rey (Coleoptera, Staphylinidae, Paederinae) from China

**DOI:** 10.3897/zookeys.447.8217

**Published:** 2014-10-16

**Authors:** Ze-Kan Lü, Li-Zhen Li

**Affiliations:** 1 Department of Biology, College of Life and Environmental Sciences, Shanghai Normal University, 100 Guilin Road, Xuhui District, Shanghai 200234, P. R. China

**Keywords:** Coleoptera, Staphylinidae, *Lobrathium*, new species, China

## Abstract

*Lobrathium jianqingi* **sp. n.** (Guangxi: Shiwanda Shan) and *Lobrathium atanggei* **sp. n.** (Yunnan: Nabanhe) from southwest China are described and illustrated.

## Introduction

In a recent checklist provided by Assing (2012), 43 species of the genus *Lobrathium* Mulsant & Rey, 1878 were reported from China. Since then, 17 additional species have been described from mainland China (Assing 2013, 2014; Li et al. 2013; Li et al. 2013a, b, c) and one name has been synonymized, thus raising the total number of species known from China to 59.

During two recent field trips we collected some *Lobrathium* specimens. Among them, two new species are recognized.

## Material and methods

The material treated in this study is deposited in the Insect Collection of Shanghai Normal University, Shanghai, China (SNUC).

Type labels are cited in their original spelling. A slash (/) is used to separate different labels.

The specimens were killed with ethyl acetate and then dried. Materials were stored in 75% ethanol; genitalia and small parts were embedded in Euparal on plastic slides that were attached to the same pin with the specimens.

Morphological studies were carried out using an Olympus SZX 16 stereoscope. A digital camera Canon EOS 7D with MP-E 65 mm Macro Photo Lens was used for the habitus photos. An Olympus CX31 microscope and a Canon G9 digital camera were used for the photos of small structures.

The measurements of various body parts are abbreviated as follows: BL – length of the body from the apical margin of the labrum to the abdominal apex; HL – length of the head from the anterior margin of the frons to the posterior constriction; HW – maximum width of the head; PL – length of the pronotum along midline; PW – maximum width of the pronotum; EL – length at the suture from the apex of the scutellum to the posterior margin of the elytra; EW – maximum width of the elytra; AL – length of the aedeagus from the apex of the ventral process to the base of the aedeagal capsule.

## Taxonomy

### Lobrathium jianqingi

Taxon classificationAnimaliaColeopteraStaphylinidae

Lü & Li sp. n.

http://zoobank.org/03A1E854-D49F-4F6A-AE4D-3F9AE603791D

Fig. 1

#### Type material.

Holotype: ♂, labelled ‘China: Guangxi Prov., Shangsi County, Shiwanda Shan N. R., 21°54'16"N, 107°54'13"E, 300–500 m, 25.IV.2011, Zhu, Peng & Zhai leg. / HOLOTYPE [red], *Lobrathium jianqingi* sp. n., Lü & Li det. 2014, SNUC’. Paratype, 1 ♀: same data as holotype.

#### Description.

**Figure 1.**
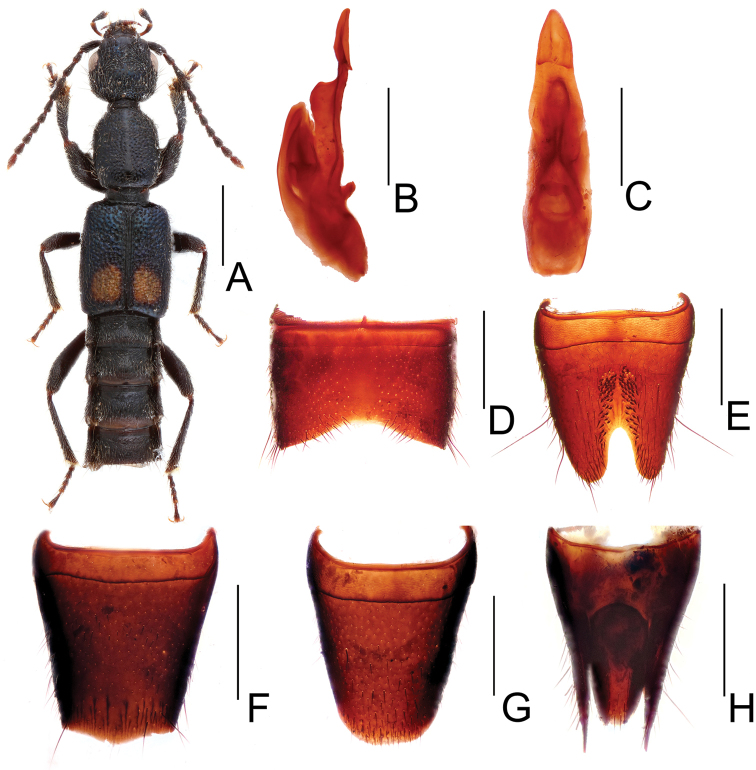
*Lobrathium jianqingi*. **A** habitus **B** aedeagus in lateral view **C** aedeagus in ventral view **D** male sternite VII **E** male sternite VIII **F** female tergite VIII **G** female sternite VIII **H** female tergites IX–X. Scale bars: **A** 1 mm, **B**–**H** 0.5 mm.

Body length 6.84–7.34 mm, length of forebody 3.73–3.78 mm. Habitus as in Fig. 1A. Coloration: body black, elytra with blue hue and subcircular large yellow spot, this spot reaching neither suture, nor lateral or posterior margins; legs black with paler tarsi, antennae blackish brown to dark brown.

Head weakly transverse (HW/HL 1.23–1.28); posterior angles broadly rounded, weakly marked; punctation dense and coarse, sparser in median dorsal portion; interstices without microsculpture. Eyes large, approximately half as long as distance from posterior margin of eye to neck in dorsal view. Antenna slender, 2.22 mm long.

Pronotum 1.13–1.24 times as long as broad and 0.90–0.97 times as wide as head, lateral margins weakly convex in dorsal view; punctation similar to that of head, midline with broad and complete impunctate band; interstices without microsculpture.

Elytra distinctly broader and longer than pronotum (EW/PW 1.33–1.39; EL/PL 1.08–1.17), humeral angles marked; punctation dense and coarse; interstices without microsculpture and glossy. Hind wings fully developed.

Abdomen narrower than elytra; punctation very fine and dense, dorsal surface nearly matt; posterior margin of tergite VII with palisade fringe.

Male: Sternites III–VI unmodified; sternite VII (Fig. 1D) strongly transverse with median impression posteriorly, this impression impunctate in the middle and on either side of middle with pubescence diagonally directed postero-mediad, posterior margin broadly and deeply concave; sternite VIII (Fig. 1E) weakly oblong, with pronounced long median impression, this impression with numerous modified, short and stout black setae, posterior excision relatively deep and almost U-shaped; aedeagus (Figs 1B, C) 1.37 mm long, ventral process long and spear-shaped apically in ventral view.

Female: Posterior margin of tergite VIII (Fig. 1F) weakly convex; posterior margin of sternite VIII (Fig. 1E) broadly convex; tergite IX (Fig. 1H) undivided anteriorly; tergite X of subovoid shape.

#### Distribution and natural history.

The type locality is situated in the Shiwanda Shan Natural Reserve, to the south of Shangsi, southern Guangxi. The specimens were found on the bank of a stream at altitudes of 300–500 m.

#### Etymology.

The species is named after Jian-Qing Zhu, one of collectors of the type specimens.

#### Remarks.

In external characters (moderate size, black body, elytra with large subcircular yellow spot), the chaetotaxy of the male sternites VII and VIII and the morphology of the aedeagus (especially long ventral process), *Lobrathium jianqingi* is most similar to *Lobrathium anatinum* Li & Li, 2013 from Guangxi. The new species is distinguished from *Lobrathium anatinum* by the more deeply concave posterior margin of the male sternite VII, the deeper posterior excision of the oblong male sternite VIII, and by the shape of the ventral process of the aedeagus in lateral view. For illustrations of *Lobrathium anatinum* see Li et al. (2013a).

### Lobrathium atanggei

Taxon classificationAnimaliaColeopteraStaphylinidae

Lü & Li sp. n.

http://zoobank.org/B28715BD-EBD3-4AF5-9EC5-ADA4909BC853

Fig. 2

#### Type material.

Holotype: ♂, labelled ‘China: Yunnan Prov., Xishuangbanna, Nabanhe N. R., alt. 700 m, 22°10'00"N, 100°39'38"E, 1.VII.2004, Liang Tang leg. / HOLOTYPE [red], *Lobrathium atanggei* sp. n., Lü & Li det. 2014, SNUC’.

#### Description.

**Figure 2.**
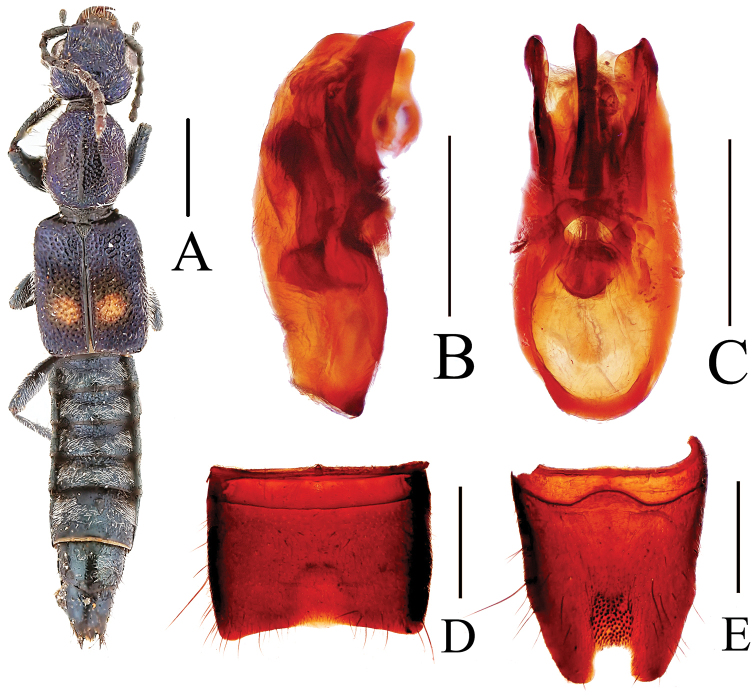
*Lobrathium atanggei*. **A** habitus **B** aedeagus in lateral view **C** aedeagus in ventral view **D** male sternite VII **E** male sternite VIII. Scale bars: **A** 1 mm, **B**–**E** 0.5 mm.

Body length 6.56 mm; length of forebody 3.11 mm. Habitus as in Fig. 2A. Coloration: body black, elytra with pronounced blue hue and small subcircular yellow spot, this spot reaching neither suture, nor lateral or posterior margins; legs black with paler tarsi, antennae blackish brown to dark yellow.

Head almost as broad as long (HW/HL 1.05), posterior angles broadly rounded, weakly marked; punctation dense and coarse, sparser in median dorsal portion; interstices without microsculpture. Eyes large, more than half as long as distance from posterior margin of eye to neck in dorsal view. Antenna slender, 2.0 mm long.

Pronotum 1.24 times as long as broad and 0.94 times as wide as head, lateral margins weakly convex in dorsal view; punctation dense and coarser than that of head, midline with broad and complete impunctate band; interstices without microsculpture.

Elytra distinctly broader and longer than pronotum (EW/PW 1.42; EL/PL 1.55), humeral angles marked; punctation dense and coarse; interstices without microsculpture and glossy. Hind wings fully developed.

Abdomen narrower than elytra; punctation very fine and dense, dorsal surface matt; posterior margin of tergite VII with palisade fringe.

Male: Sternites III–VI unmodified; sternite VII (Fig. 2D) strongly transverse, with pronounced median impression posteriorly, with sparse unmodified pubescence, and with broadly concave posterior margin; sternite VIII (Fig. 2E) weakly oblong, with deep median impression posteriorly, this impression with numerous modified, very short and stout black setae, posterior excision deep and almost U-shaped; aedeagus (Figs 2B, C) 1.10 mm long, ventral process somewhat asymmetric and of distinctive shape.

Female: unknown.

#### Distribution and natural history.

The type locality is situated in the Nabanhe Natural Reserve, to the northwest of Xishuangbanna, southwestern Yunnan. The holotype was found on the bank of a stream at an altitude of 700 m.

#### Etymology.

The species is named after Liang Tang (nickname “Atangge”), who collected the holotype.

#### Remarks.

In external characters (black body, weakly transverse head, slender pronotum, elytra with small subcircular spot), as well as the shape and chaetotaxy of the male sternites VII and VIII, *Lobrathium atanggei* is similar to *Lobrathium ablectum* Assing, 2012 from Hubei. The new species is readily distinguished from *Lobrathium ablectum* by the somewhat smaller size, the pronounced blue hue of the body and a stout ventral process of the aedeagus. Regarding the morphology of the aedeagus (robust and with short ventral process), however, the new species is most similar to *Lobrathium quadrum* Li, Solodovinikov & Zhou, 2013 from Sichuan. It is distinguished from *Lobrathium quadrum* by the pronounced blue hue of the body; the yellowish spot on elytra, reaching neither suture, nor lateral or posterior margins; the modifications of the male sternites VII–VIII (sternite VII with pronounced median impression posteriorly, sternite VIII with numerous modified, very short and stout black setae; deep posterior excision). For illustrations of *Lobrathium ablectum* and *Lobrathium quadrum* see Assing (2012) and X.-Y. Li et al. (2013), respectively.

## Supplementary Material

XML Treatment for Lobrathium jianqingi

XML Treatment for Lobrathium atanggei
